# 172. The First Proof-of-Concept Clinical Trial of an HIV-1 Allosteric Integrase Inhibitor, Pirmitegravir (STP0404)

**DOI:** 10.1093/ofid/ofaf695.002

**Published:** 2026-01-11

**Authors:** Moti Ramgopal, Paul Benson, John C Williamson, Mezgebe Berhe, Peter Ruane, Beata Casanas, Edwin DeJesus, Christopher Lucasti, Xue Meng, Uk-Il Kim, Song Hyun Kim, Moo Je Sung

**Affiliations:** Midway Immunology and Research Center, Fort Pierce, Florida; Be Well Medical Center, Berkley, MI; Wake Forest School of Medicine, Winston-Salem, NC; North Texas Infectious Diseases Consultants, Dallas, Texas; Ruane Clinical Research, Los Angeles, CA, USA, Los Angeles, California; University of South Florida, Tampa, Florida; Orlando Immunology Center, University of Central Florida College of Medicine, Orlando, FL; South Jersey Infectious Disease, Somers Point, New Jersey; ST Pharm Co., Ltd., Seoul, Seoul-t'ukpyolsi, Republic of Korea; ST Pharm Co., Ltd., Seoul, Seoul-t'ukpyolsi, Republic of Korea; ST Pharm Co., Ltd., Seoul, Seoul-t'ukpyolsi, Republic of Korea; ST Pharm Co., Ltd., Seoul, Seoul-t'ukpyolsi, Republic of Korea

## Abstract

**Background:**

Pirmitegravir (STP0404) is the first HIV-1 allosteric integrase inhibitor (ALLINI) to enter a proof-of-concept (POC) clinical trial. It has demonstrated potent *in vitro* anti-HIV-1 activity with *in vitro* distinct resistance profile compared to existing catalytic-site integrase inhibitors (INSTIs), and favorable safety and pharmacokinetics (PK) profiles in Phase 1 and nonclinical studies.

**Methods:**

This Phase 2a, randomized, double-blinded, placebo-controlled POC trial (NCT05869643) evaluated the antiviral activity, safety, tolerability and PK characteristics of STP0404 in a design in adults living with HIV-1 who were antiretroviral therapies (ARTs)-naive or had limited prior ARTs exposure. Participants received STP0404 (200 mg, 400 mg or 600 mg) or placebo once daily for 10 days. The interim analysis results of the first 2 completed cohorts (200 mg and 400 mg) are presented below.

**Results:**

Sixteen participants (ages 20-42, including one female at birth) were enrolled. Significant reductions in plasma HIV-1 RNA levels were observed from baseline to Day 11 in both STP0404 treatment arms (*p*< 0.0001), with a mean decrease of 1.184 to 1.557 log_10_ copies/mL. Three out of 16 treatment-emergent adverse events (AEs) reported in one participant in Cohort 1 (200 mg) were possibly related to the study drug. No severe AEs, serious adverse events (SAEs), or discontinuations due to AEs were observed up to interim analysis. All AEs resolved or recovered. The PK profile of STP0404 was linear but less than dose-proportional across the administered dose range. Median peak concentrations were achieved approximately 4.5 to 5.5 hours post-dose, with mean terminal half-lives of 13.7 and 11.6 hours for respective 200 and 400 mg dose levels. Minimal or no apparent accumulation was observed following repeated dosing on Day 10 for either dose levels.

**Conclusion:**

Pirmitegravir (STP0404) demonstrated significant antiviral efficacy, a dose-independent PK profile at 200 mg and 400 mg, and was very well tolerated. As a novel ALLINI, it is anticipated to provide new therapeutic options while complementing existing antiretroviral regimens in the near future. Results of Cohort 3 (600 mg) are expected in early 2026.

**Disclosures:**

All Authors: No reported disclosures

